# Multiple sex partner behavior in female undergraduate students in China: A multi-campus survey

**DOI:** 10.1186/1471-2458-9-305

**Published:** 2009-08-22

**Authors:** Hong Yan, Weiqi Chen, Haocheng Wu, Yongyi Bi, Miaoxuan Zhang, Shiyue Li, Kathryn L Braun

**Affiliations:** 1 School of Public Health of Wuhan University, Wuhan, Hubei, PR China; 2 Office of Public Health Studies, John A. Burns School of Medicine, University of Hawaii, Honolulu, HI, USA

## Abstract

**Background:**

China is realizing increases in women engaged in premarital sex and multiple sex partner behavior. Our aim was to examine prevalence and determinants of multiple sex partner behavior among female undergraduates in China.

**Methods:**

Anonymously completed questionnaires were received from 4,769 unmarried female undergraduates, recruited using randomized cluster sampling by type of university and students' major and grade. Items captured demographic, family, peer and work influence, and student factors (major, academic performance, and sex-related knowledge and attitudes). To examine risk factors for sexual behaviors, we used multi-level logistic regression, yielding odds ratios (OR) and 95% confidence intervals (95% CI).

**Results:**

Of 4,769 female students, 863 (18.10%) reported ever having sexual intercourse, and 5.31% reported having multiple sex partners (29.32% of all women having sexual intercourse). Several demographic, family, peer and work influences, and student factors (including major, performance, knowledge, and attitude toward sex) were risk factors for ever having sex. However, risk factors for multiple sex partners only included working in a place of entertainment, having current close friends that were living with boyfriends, poor academic performance, and positive attitudes toward multiple partners. These women also were more likely to practice masturbation, start having sex at a younger age, have sex with married men and/or men not their "boyfriends" at first coitus, and not use condoms consistently.

**Conclusion:**

A small but important subset of Chinese female undergraduates is engaged in unprotected sex with multiple sex partners. Interventions need to target at risk women, stressing the importance of consistent condom use.

## Background

Since the start of open-door policies in the 1970s and the economic reforms of the 1980s, China has experienced dramatic social changes associated with rapid economic reform and growth. Traditional attitudes towards sex, marriage, and family have changed. Premarital sex, formerly an unacceptable behavior, is being reported by a growing number of young people, including college undergraduates, about 11.3% in 2000, up from 9.8% in 1990 [1-4]. In the last decade, research on the sexual behavior of Chinese university students has shown that 9–16% of students report that they are sexually experienced [5]. Attitude toward premarital sex may impact behavior, and an investigation in China showed that 44.0% of undergraduates thought that premarital sex was acceptable [6].

The prevalence of sexually transmitted diseases (STDs) also has increased dramatically over the past few decades [7,8]. Sexually transmitted HIV infections, for example, have increased in prevalence from 5.5% in 1997 to 19.8% in 2003 [9]. In recent years, HIV infection through sexual transmission is growing the fastest, from 7.2% in 2002 to 43.6% in total infections by the end of 2005 [10]. Sexually active adolescents and young adult are particularly vulnerable to HIV/STDs infection, and the United Nations estimates that about half of new HIV infections worldwide occur among people aged 15–24 years [11]. In developing countries, adolescents (15–24 years old) make up only 25% of the sexually active population, but represent almost 50% of all new acquired STDs [12]. In China, more than 60% of all HIV infections are among people aged 15–29 years [3].

Although HIV in China is still concentrated among injection drug users, former plasma donors, sex workers and their clients, and men who have sex with men (MSM) [13], there were indications that the disease is spreading from these high-risk groups to the general population [14,15]. High-risk heterosexual behavior is a contributive factor [16]. It is estimated that half of the new HIV infections in China in 2005 occurred through unprotected heterosexual encounters [11]. A 2002 survey of students aged 17 to 28 in China estimated that 14% of undergraduates were sexually active, 24% of responding students considered themselves to be at a moderate to very high risk of contracting HIV, and 40% of sexually active students reported never using condoms [17].

Safe sexual behaviors include having a single sex partner and using condoms in every sexual encounter, and these behaviors also reduce risk of HIV/STDs. Having multiple sex partners is a significant behavioral risk factor for HIV/STDs [18]. Adolescents typically engage in short-lived relationships that make them more likely than adults to have sex with multiple partners, thereby placing them at greater risk for contracting HIV/STDs. As the number and variety of new sex partners increases, so does the risk of exposure to individuals infected with STDs [19]. The presence of an STD greatly increases a person's likelihood of acquiring or transmitting HIV through sexual contact [20]. Most importantly, those who have multiple sex partners are less likely to use condoms during sex [18,19,21].

The risk of adverse health outcomes from multiple sex partners is greater for females than males, as it has direct influence on reproductive health [22]. Untreated STDs may make females susceptible to cervical dysplasia and pelvic inflammatory disease, which can lead to infertility, tubal pregnancies, and compromised fetal health [23]. Adolescent girls who have multiple partners may also be vulnerable to dating violence [24].

There have been some studies on the sexual behavior of undergraduates [4,17,25-29]. However, few studies have explored multiple sex partner behavior and associated risk factors among female undergraduates in China. Understanding these risk factors can help health care practitioners and health educators develop messages and interventions to reduce young peoples' risk of infection with HIV and other STDs.

The present study was conducted in Wuhan, China, a city of about 8 million on the Yangzi River in Hubei Province west of Shanghai. Female undergraduates were recruited from 16 campuses to complete anonymous, self-administered questionnaires. Data were used to examine the prevalence and determinants of multiple sex partner behavior in this population.

## Methods

### Sample

Of 43 universities in Wuhan, 16 were randomly selected according to type–2 out of 4 state universities, 10 of 25 universities under individual ministries, and 4 of 12 local universities. To assure adequate proportions of students across the four years of college and in four majors (literature and history, science and technology, medicine, and art), sampling on each campus was purposive. For example, because few colleges offer art, we endeavored to recruit all female art students on campuses that offered this. To be eligible for the study, female undergraduates needed to be unmarried and needed to provide consent.

The recruitment period was December 2005 and April 2006. Investigators enrolled 5,076 female undergraduates across 16 sites. Enrollees completed questionnaires in classrooms with seating arranged to assure privacy. Students were informed about the study and its purpose, assured that participation was voluntary, and reminded that the survey was anonymous and not to put their name on it. All participants were asked to consent to participate. Those who consented were given 30 minutes to complete the questionnaire, although most women took less time to complete it. When finished, students were instructed to insert their questionnaire into a locked box.

Marked questionnaires were returned from 4,923 (97%) enrollees; 3% of the enrollees either changed their minds about participating and did not provide consent, or they were unable or unwilling to complete the questionnaire after providing consent. Returned questionnaires were reviewed by research staff for completeness and consistency, and 154 compromised questionnaires were discarded. The remaining 4,769 questionnaires represented 94% of the initial sample.

### Measures

The questionnaire was developed from the relevant literature, which identified several factors associated with engaging in premarital sex in China. These include knowledge about sex, attitudes toward sex, attitudes of family and peers toward sex, and age [17,25,28,29]. Also, preliminary studies in China suggested that students who major in the arts (vs. the sciences) are more likely to engage in premarital sex [29].

Thus, data were collected in four areas: 1) demographics; 2) family, peer, and work influences; 3) current student variables (knowledge, attitudes, and situation in college); and 4) sex-related behaviors. Demographic items included age, nationality (Han vs. ethnic minority), home location (eastern coastal regions, central areas, or western areas), and family economic status (poor, average, or rich).

Family, peer, and work influences included parents' disciplinary style (strict, general, or relaxed), if the parents were divorced, if the woman was an only child, if middle-school close friends fell in love, if current close friends lived with boyfriends, and if the respondent was employed in a place for entertainment, such as a pub, club and disco (1 = yes, 0 = no). We also asked students to indicate the attitudes of their parents and middle-school close classmates and friends toward premarital sex. Response options were approve of this behavior, understand this behavior in others, and disapprove of this behavior. For the multivariate analysis, attitude responses were collapsed (1 = approve or understand and 0 = disapprove).

Current student variables included major (literature and history, science and technology, medicine, or art), grade (freshman, sophomore, junior, or senior), academic performance (excellent, average, poor), and feelings at school (generally happy, ordinary, confused, depressed, anxious, and/or pressured). Sex-related knowledge was measured using a 27 items regarding reproduction (e.g., Do you know the stages of normal menstrual cycle?), contraceptives (e.g., Do you know how to use condom?), STDs (e.g., Is gonorrhea a sexually transmitted disease?), and AIDs (e.g., Is sexual intercourse a route of transmission of AIDs?). Response options were yes, no, and don't know; correct answers were coded 1, and other responses coded 0. Coded responses were summed and converted to a 100-point scale by multiplying by 0.037(1/27). Higher scores reflected better sex-related knowledge. Attitudes towards premarital sex and multiple sex partner were measured using the questions "Do you approve of having sexual intercourse before marriage?" and "How do you feel about having multiple sex partner?" As noted earlier, response options were approve, understand, or disapprove of this behavior, with responses collapsed for multivariate analysis (1 = approve or understand and 0 = disapprove).

For behavioral items, respondents were asked if they ever had sexual intercourse (1 = yes, 0 = no). If a respondent answered "yes," she was asked to provide information on age of first coitus and lifetime number of sex partners. In the multivariate analysis, responses were grouped into two categories: single vs. multiple sex partners. Respondents who had had sex were asked if the partner was married to someone else (1 = yes, 0 = no) or if their first partner was a boyfriend (1 = no, 0 = yes). Also asked were questions about masturbation (1 = yes, 0 = no) and condom practices (sometimes or never = 1, always or almost always = 0).

The questionnaire was pretested with 109 medical sciences majors. In addition to readability and time for completion, we examine test-retest reliability. Students were surveyed at two time points, 10 days apart, with student self-assigned identification numbers so surveys could be linked across time points. In all, 101 pairs of completed questionnaires were received. Knowledge scores across the two time points were 34.29 ± 8.76 and 33.27 ± 7.69, respectively. The scores were highly correlated (0.735, *P *< 0.001), and not significant different (t = 1.012, *P *= 0.40). Nor were there significant differences over time in proportions reporting sexual intercourse (*x*^2 ^= 0.149, *P *= 0.70), or disapproving of premarital sex (*x*^2 ^= 0.63, *P *= 0.73) and multiple sex partner (*x*^2 ^= 3.071, *P *= 0.215).

### Statistical Analysis

The data was initially subject to preliminary descriptive analysis using statistical software SPSS 11.5. Significant variables were included in multi-level logistic regression models to examine relative influences of demographics, family/peer/work influences, and current student factors on the two dichotomous dependent variables–ever having sex and multiple sex partners. Because of hierarchical data (students were recruited from different universities), the multi-level logistic regression models were adjusted for clustering of students (level 1) within university (level 2) in this study. To do this, we fitted a baseline variance component or empty model (no independent variables) followed by the model with demographics variables (Model 1). Model 2 expands Model 1 by including family/peer/work influences. The final model (Model 3) expands Model 2 by introducing current student factors. The significance of the fixed and random parameter variance estimates (university variance) was assessed using the Wald joint *x*^2 ^test statistic. The proportion of the university variance explained for each model was estimated as the difference in university variance between baseline (empty model) and Model 1 or Model 2 or Model 3 divided by the university variance for the baseline model [30]. All multi-level models were performed with MLwiN version 2.02.

### Ethics

The study was approved by the Medical Ethics Committee of Wuhan University. The written consent was obtained from all students who participates the study.

## Results

### Sample characteristics

Of the 4,769 female undergraduates in the sample, the mean age was 20.35 years (range 16–27), 88.43% were of Han nationality, and 74.40% were from the central areas of China (Table 1). About 10% of students reported that parents were relatively rich, that 5.54% were divorced, that 16.67% had a strict disciplinary style, and that 46.45% were disapproving of premarital sex. Only 41% were from one-child families. About 60% reported that close middle-school classmates and friends were disapproving of premarital sex, but 28% had middle-school friends who had fallen in love. Currently, only 8.32% had close friends living with boyfriends, and only 6.46% worked at a place of entertainment. Using stratified sampling, we achieved adequate numbers across the four college majors and the four years at school. About 26% reported excellent academic performance, while 10.65% reported poor performance. About 28% reported feeling generally happy, but percentages of students feeling anxious, depressed, pressured, or confused ranged from 12.39% to 48.06%.

**Table 1:** Characteristics of the full sample and those who had and never had sex

Variables	Full sample (n = 4,769)	Ever had sex (n = 863)	Test of signif T-test or OR (95% CI)
**Demographic Variables**			
Mean age^a^	20.35(SD = 1.49)	20.99(SD = 1.40)	14.10^d^
Nationality Han (vs. minority)	4,217 (88.43)	779 (90.27)	0.79(0.62–1.01)
Home location			
Eastern coastal regions	561 (11.76)	107 (12.40)	1.00(reference)
Central areas	3,548 (74.40)	662 (76.71)	0.97(0.78–1.22)
Western areas	660 (13.8)	94 (10.89)	0.71(0.52–0.95)^c^
Parents' economic status			
Poor	2,142 (44.92)	212 (24.57)	1.00(reference)
Average	2,158 (45.25)	455 (52.72)	2.43(2.04–2.90)^d^
Rich	469 (9.83)	196 (22.71)	6.54(5.18–8.24)^d^
**Family, peer, and work variables**			
Parents' disciplinary style			
Strict	795 (16.67)	195 (22.60)	1.00(reference)
Average	2,327 (48.79)	434 (50.29)	0.71(0.58–0.86)^d^
Relaxed	1,647 (34.54)	234 (27.11)	0.51(0.41–0.63)^d^
Only one child(yes)^b^	1,954(40.97)	468(54.23)	1.93(1.66–2.24)^d^
Parents divorced(yes)^b^	264(5.54)	86(9.97)	2.32(1.77–3.03)^d^
Parents disapproving of premarital sex (yes)^b^	2,215 (46.45)	416 (48.20)	1.09(0.94–1.26)
Middle school close classmates and friends disapproving of premarital sex (yes)^b^	2,796(58.63)	472(54.69)	0.82(0.71–0.95)^c^
Middle school close friends falling in love (yes)^b^	1,338(28.06)	366(42.41)	2.22(1.91–2.59)^d^
Current close friends living with boyfriend (yes)^b^	397 (8.32)	224 (25.96)	7.56(6.10–9.38)^d^
Work at place of entertainment (yes)^b^	308 (6.46)	138 (15.99)	4.18(3.30–5.31)^d^
**Current student variables**			
Major			
Literature and history	1,862 (39.04)	323 (37.43)	1.00(reference)
Science and technology	1,167 (24.47)	157 (18.19)	0.74(0.60–0.91)^d^
Medical science	985 (20.65)	118 (13.67)	0.65(0.52–0.81)^d^
Art	755 (15.83)	265 (30.71)	2.58(2.13–3.12)^d^
Grade			
Freshman	1,373 (28.79)	103 (11.94)	1.00(reference)
Sophomore	1,195 (25.06)	157 (18.19)	1.87(1.44–2.42)^d^
Junior	1,309 (27.45)	361 (41.83)	4.70(3.71–5.94)^d^
Senior	892 (18.70)	242 (28.04)	4.59(3.58–5.89)^d^
Academic performance			
Excellent	1,243 (26.06)	210 (24.33)	1.00(reference)
Medium	3,018 (63.28)	546 (63.27)	1.09(0.91–1.29)
Poor	508 (10.65)	107 (12.40)	1.31(1.01–1.70)^c^
Feelings in college			
Generally happy(yes)^b^	1,324(27.76)	236(27.35)	0.98(0.83–1.15)
Average or ordinary(yes)^b^	1,523(31.94)	264(30.59)	0.93(0.79–1.09)
Confused(yes)^b^	2,292(48.06)	437(50.64)	1.13(0.98–1.31)
Depressed(yes)^b^	932(19.54)	195(22.60)	1.26(1.05–1.50)^c^
Anxious(yes)^b^	591(12.39)	118(13.67)	1.15(0.93–1.43)
Pressured(yes)^b^	1,328(27.85)	235(27.23)	0.96(0.82–1.14)
Score in sex-related knowledge ^a^	42.58(SD = 12.99)	51.01(SD = 11.68)	22.12^d^
Approve/accept premarital sex (yes)^b^	2,837 (59.49)	790 (91.54)	9.83(7.67–12.59)^d^
Approve/accept multiple sex partners (yes)^b^	547 (11.47)	164 (19.00)	2.16(1.77–2.64)^d^

^a ^Independent-samples t-test. ^b ^Dichotomous variables with 0 (condition absent) as reference group. ^c ^p < 0.05 ^d ^p < 0.01

The average score on the sex-related knowledge scale was 42.58 ± 12.99 (out of 100). Only 8.81% scored 60 or above, and 42.19% scored less than 40. Almost 60% had favorable attitudes toward premarital sex, including 9.81% that approved and 49.67% who understood the behavior in others. However, only 11.47% of the entire sample reported approval or understanding of having multiple sex partners.

Also shown in Table 1 are the characteristics of the 863 students who had had sex. Compared to the general sample, they tended to be older, and not be from western areas. They were more likely to be only children, from richer families, and to have parents who were divorced and strict in their disciplinary style. They were less likely to have middle-school close classmates and friends who disapproved of premarital sex and more likely to have middle-school friends who fell in love. They were more likely to have current close friends that lived with boyfriends and to themselves work at places of entertainment. Art majors were more likely to report having sex than students in other majors, and greater percents reported poor academic performance and feeling depressed. Mean sex-related knowledge scores were higher, by almost 10 points, as were proportions approving or understanding of premarital sex (91.54% vs. 59.49%) and multiple sex partners (19.00% vs. 11.47%).

Table 2 focuses on the 863 students who had had sex and examines differences between those with and without multiple sex partners. Many of the same variables were significant, e.g., not being from western states; being from richer, one-child, and/or divorced families; having middle-school, current friend, and work influences that supported sex; majoring in art; and poor academic performance. However, more students with multiple sex partners report feeling anxious. Although sex-knowledge scores were significantly higher, they were only 2 points higher among those with multiple sex partners. Understandably, greater proportions approved/accepted premarital sex and multiple sex partner behavior.

**Table 2:** Characteristics of those students with single vs. multiple sex partners

Variables	Ever had sex (n = 863)	Multiple sex partners (n = 253)	Test of signif T-test or OR (95% CI)
**Demographic Variables**			
Mean age ^a^	20.99(SD = 1.40)	21.09(SD = 1.31)	0.92
Nationality Han (vs. minority)	779 (90.27)	231 (91.30)	0.85(0.51–1.43)
Home location			
Eastern coastal regions	107 (12.40)	37 (14.62)	1.00(reference)
Central areas	662 (76.71)	196 (77.47)	0.76(0.49–1.18)
Western areas	94 (10.89)	20 (7.91)	0.49(0.26–0.94)^c^
Parents' economic status			
Poor	212 (24.57)	38 (15.02)	1.00(reference)
Average	455 (52.72)	131 (51.78)	1.79(1.19–2.70)^d^
Rich	196 (22.71)	84 (33.20)	3.24(2.05–5.12)^d^
**Family, peer, and work variables**			
Parents' disciplinary style			
Strict	195 (22.60)	60 (23.72)	1.00(reference)
Average	434 (50.29)	131 (51.78)	1.00(0.68–1.45)
Relaxed	234 (27.11)	62 (24.51)	0.80(0.52–1.23)
Only one child(yes)^b^	468(54.23)	156(61.66)	1.55(1.14–2.09)^d^
Parents divorced(yes)^b^	86(9.97)	37(14.62)	1.90(1.20–3.03)^d^
Parents disapproving of premarital sex (yes)^b^	416 (48.20)	113 (44.66)	0.82(0.61–1.11)
Middle-school close classmates and friends disapproving of premarital sex (yes)^b^	472(54.69)	123(48.62)	0.71(0.52–0.95)^c^
Middle-school close friends falling in love (yes)^b^	366(42.41)	132(52.17)	1.73(1.28–2.33)^d^
Current close friends living with boyfriend (yes) ^b^	224 (25.96)	104 (41.11)	2.73(1.97–3.78)^d^
Work at place of entertainment (yes)^b^	138 (15.99)	70 (27.67)	2.88(1.97–4.20)^d^
**Current student variables**			
Major			
Literature and history	323 (37.43)	80 (31.62)	1.00(reference)
Science and technology	157 (18.19)	26 (10.28)	0.56(0.34–0.91)^c^
Medical science	118 (13.67)	32 (12.65)	1.11(0.68–1.81)
Art	265 (30.71)	115 (45.45)	2.02(1.41–2.89)^d^
Grade			
Freshman	103 (11.94)	18 (7.11)	1.00(reference)
Sophomore	157 (18.19)	43 (17.00)	1.37(0.72–2.58)
Junior	361 (41.83)	122 (48.22)	1.85(1.04–3.27)^c^
Senior	242 (28.04)	70 (27.67)	1.46(0.80–2.65)
Academic performance			
Excellent	210 (24.33)	50 (19.76)	1.00(reference)
Medium	546 (63.27)	162 (64.03)	1.43(0.98–2.07)
Poor	107 (12.40)	41 (16.21)	1.99(1.20–3.31)^d^
Feelings in college			
Generally happy(yes)^b^	236(27.35)	65(25.69)	0.89(0.63–1.24)
Average or ordinary(yes)^b^	264(30.59)	66(26.09)	0.76(0.54–1.06)
Confused(yes)^b^	437(50.64)	132(52.17)	1.11(0.82–1.50)
Depressed(yes)^b^	195(22.60)	65(25.69)	1.34(0.94–1.90)
Anxious(yes)^b^	118(13.67)	48(18.97)	1.79(1.19–2.70)^d^
Pressured(yes)^b^	235(27.23)	74(29.25)	1.12(0.80–1.56)
Score in sex-related knowledge ^a^	51.01(SD = 11.68)	53.09(SD = 11.59)	2.75^d^
Approve/accept premarital sex (yes)^b^	790 (91.54)	247 (97.63)	4.34(1.84–10.25)^d^
Approve/accept multiple sex partners (yes)^b^	164 (19.00)	82 (32.41)	3.25(2.26–4.68)^d^
			

^a ^Independent-samples t-test. ^b ^Dichotomous variables with 0 (condition absent) as reference group. ^c ^p < 0.05 ^d^p < 0.01

Sexual behavior variables are shown in Table 3. Of 4,769 female students, 18.10% reported ever having sexual intercourse, and 5.31% reported having multiple sex partners. Of students who had engaged in sexual intercourse, 63.18% reported having a single sexual partner. 29.32% of them reported having multiple partners. Those with multiple partners were more likely to report masturbating. They were younger at first coitus (18.74 vs. 19.27). The prevalence of multiple sex partners among those who first had sex at age 18 or younger were 46.18% (115/249), while 25.09% (137/546) among females who first had sex at age 19 or older. Those with multiple partners were also more likely to have had sex with a married man or someone not their boyfriend at first coitus, and to report inconsistent condom use (38.61% vs. 29.12%).

**Table 3:** Sexual behavior variables for full sample, those who ever had sex, and those with multiple sex partners

Sexual behavior variables	Full sample (n = 4,769)	Ever had sex (n = 863)	Multiple sex partners (n = 253)	Test of significance^a ^ T-test or OR (95% CI)
Ever had intercourse (yes)	863 (18.10)	863 (100.0)	253 (100.0)	
Had multiple sex partners (yes)	253 (5.31)	253 (29.32)	253 (100.0)	
Practice masturbation (yes)	1,137(23.84)	292 (33.84)	99 (39.13)	1.39 (1.02–1.90)^c^
Age at first coitus ^b^		19.27(SD = 1.74)	18.74(SD = 1.60)	6.4^d^
Partner at first coitus not being boyfriend (yes)		40 (4.63)	19 (7.51)	2.88 (1.44–5.77)^d^
Had sex with married man (yes)		48 (5.56)	30 (11.86)	4.75 (2.51–9.01)^d^
**Inconsistent **condom use during sex		108 (29.12)	78(38.61)	2.01 (1.39–2.91)^d^

^a ^Testing difference between those with (n = 253) and without (n = 610) multiple sex partners. ^b ^Independent-samples t-test. ^c ^p < 0.05 ^d ^p < 0.01

### Factors associated with ever having sex

All significant variables in Table 1 were entered into regression analysis (Table 4). The between-university variance (i.e. the random intercept) was statistically significant in Model 1 (meaning that there were significant differences in ever having sex between universities after adjusting for demographic variables), and demographic variables explained 63.40% of the between-university variance (Model 1). However, between-university variance was not significant in Models 2 and 3. The addition of family/peer/work influences explained about 90% of the between-university variance (Model 2). In Model 3, current student factors only explained about 7% of the variation. This suggests that the demographic variables and family/peer/work influences included in Model 2 were important in explaining the variation.

**Table 4:** Predictors of ever having sexual intercourse in female undergraduate students (n = 4,769)

Explanatory variables	Empty Model	Model 1 Demographics OR (95% CI)	Model 2 Demos + influ OR (95% CI)	Model 3 All factors OR (95% CI)
**Demographic Variables**				
Age (continuous)		1.52(1.43–1.61)^d^	1.46(1.38–1.55)^e^	1.27(1.16–1.39)^e^
Home location^a^				
Eastern coastal regions		1.00	1.00	1.00
Central areas		1.33(1.03–1.72)^e^	1.25(0.96–1.63)	1.35(1.02–1.80)^e^
Western areas		1.18(0.85–1.64)	1.03(0.73–1.45)	0.95(0.66–1.36)
Parents' economic status^a^				
Poor		1.00	1.00	1.00
Average		2.15(1.77–2.60)^f^	1.92(1.57–2.34)^f^	1.48(1.19–1.83)^f^
Rich		4.93(3.77–6.45)^f^	3.79(2.86–5.01)^f^	2.76(2.03–3.75)^f^
**Family, peer, and work influences**				
Parents' disciplinary style^a^				
Strict			1.00	1.00
Average			0.76(0.61–0.95)^e^	0.75(0.59–0.94)^e^
Relaxed			0.65(0.51–0.82)^f^	0.58(0.45–0.75)^f^
Only one child(yes)^b^			1.16(0.97–1.39)	0.89(0.73–1.08)
Parents divorced(yes)^b^			1.91(1.40–2.60)^f^	1.64(1.17–2.31)^f^
Middle-school close classmates and friends disapproving of premarital sex (yes)^b^			0.91(0.76–1.08)	1.01(0.83–1.21)
Middle-school close friends falling in love (yes)^b^			1.50(1.25–1.79)^f^	1.10(0.90–1.33)
Current close friends living with boyfriend (yes) ^b^			3.40(2.66–4.35)^f^	2.48(1.90–3.24)^f^
Work at place of entertainment (yes)^b^			2.37(1.78–3.15)^f^	2.11(1.54–2.88)^f^
**Current student factors**				
Major^a^				
Literature and history				1.00
Science and technology				1.32(1.03–1.81)^e^
Medical science				0.32(0.23–0.45)^f^
Art				1.41(0.97–2.05)
Year in school (continuous)				1.12(0.98–1.27)
Academic performance^a^				
Excellent				1.00
Medium				1.37(1.11–1.69)^f^
Poor				1.92(1.39–2.64)^f^
Feelings depressed in college(yes) ^b^				1.02(0.97–1.08)
Score in sex-related knowledge (continuous) ^b^				1.07(1.06–1.08)^e^
Approve/accept premarital sex (yes)^b^				5.00(3.83–6.52)^f^
Approve/accept multiple sex partners (yes)^b^				0.97(0.76–1.24)
**Between university variance(SE^c^)**	0.429(0.175)	0.157(0.074)	0.045(0.029)	0.017(0.018)
**Explained variance^d ^(%)**		63.40	89.51	96.04

^a ^The first category was used as reference group. ^b ^Dichotomous variables with 0 (condition absent) as reference group. ^c ^Standard error. ^d ^Explained 'between university' variance using the variance in the empty model as reference. ^e ^p < 0.05 ^f ^p < 0.01

Age, home location, and parents' economic status are significant demographic factors. Under influences, those more likely to engage in premarital sex were from divorced families, have parents with a strict disciplinary style, have current friends who lived with boyfriends, and work at a place of entertainment. Those students having higher sex-related knowledge and more approving attitudes toward sex were more likely to have premarital sex.

### Factors associated with multiple sex partners

Risk factors of multiple partners are shown in Table 5. The between-university variance (random intercept) was not statistically significant in four models, indicating that there were not significant differences in multiple sex partners between universities after adjusting for demographic variables, family/peer/work influences and current student factors. Demographic variables (Model 1) explained 14.02% of the between-university variance, while demographic variables and family/peer/work influences (Model 2) explained approximately 55% of the between-university variance. The final model including all variables, however, only explained about 3% of the between-university variance. This may be a result of methodological limitations. The addition of current student factors may have prevented the detection of associations. These results suggest that family/peer/work influences are important in explaining the variation or that multiple sex partner behavior is possibly influenced by factors other than those measured in this study.

**Table 5:** Predictors of multiple sex partner behavior in female undergraduate students (n = 863)

Explanatory variables	Empty Model	Model 1 Demos OR (95% CI)	Model 2 Demos + influ OR (95% CI)	Model 3 All factors OR (95% CI)
**Demographic Variables**				
Home location^a^				
Eastern coastal regions		1.00	1.00	1.00
Central areas		0.70(0.42–1.40)	0.68(0.41–1.13)	0.77(0.45–1.31)
Western areas		0.62(0.31–1.22)	0.58(0.29–1.16)	0.54(0.26–1.12)
Parents' economic status				
Poor		1.00	1.00	1.00
Average		1.44(0.92–2.24)	1.36(0.86–2.14)	1.08(0.66–1.77)
Rich		2.27(1.36–3.79)^f^	1.92(1.13–3.26)^e^	1.44(0.81–2.57)
**Family, peer, and work influences**				
Only one child(yes)^b^			1.07(0.76–1.51)	1.11(0.76–1.60)
Parents divorced(yes)^b^			1.65(0.99–2.73)	1.36(0.80–2.33)
Middle-school close classmates and friends disapproving of premarital sex (yes)^b^			0.87(0.62–1.21)	0.94(0.66–1.32)
Middle-school close friends falling in love (yes)^b^			1.27(0.91–1.78)	1.20(0.84–1.72)
Current close friends living with boyfriend (yes) ^b^			1.98(1.38–2.83)^f^	1.58(1.07–2.32)^e^
Work at place of entertainment (yes)^b^			2.21(1.45–3.38)^f^	2.04(1.30–3.20)^f^
**Current student factors**				
Major^a^				
Literature and history				1.00
Science and technology				0.76(0.42–1.36)
Medical science				1.52(0.70–3.30)
Art				1.99(0.72–5.49)
Year in school (continuous)				1.04(0.85–1.28)
Academic performance^a^				
Excellent				1.00
Medium				1.42(0.94–2.15)
Poor				2.91(1.59–5.33)^f^
Feelings anxious in college(yes)^b^				1.16(1.00–1.36)
Score in sex-related knowledge (continuous)				1.02(1.00–1.04)
Approve/accept premarital sex (yes)				2.46(0.96–6.33)
Approve/accept multiple sex partners (yes)^b^				2.59(1.73–3.88)^f^
**Between university variance(SE^c^)**	0.264(0.157)	0.227(0.144)	0.118(0.095)	0.257(0.162)
**Explained variance^d ^(%)**		14.02	55.30	2.65

^a ^The first category was used as reference group. ^b^Dichotomous variables with 0 (condition absent) as reference group. ^c^Standard error. ^d ^Explained 'between school' variance using the variance in the empty model as reference. ^e ^p < 0.05 ^f ^p < 0.01

Compared with ever having sex, fewer variables distinguish women with single and multiple sex partners. These include working in a place of entertainment, having current close friends living with boyfriends, poor academic performance, and positive attitudes toward multiple partners.

## Discussion

Most female undergraduates in this study thought premarital sex was acceptable, and 18.10% were sexually active. This prevalence is higher than reported in previous studies in China [4,5,17,27]. Of students who had engaged in sexual intercourse, the majority (63.18%) reported having a single sexual partner. However, 29.32% of them reported having multiple partners. Certainly, compared with the United States (US) and other developed countries, the prevalence of multiple sex partner behavior among Chinese female undergraduates is relatively low, possibly due to their conservative attitude to multiple sex partner behavior (only 11.47% approve of or accept this behavior). According to CDC's YRBS in 2007, 14.9% of US high school students had had sexual intercourse with four or more persons during their life [31]. In another study of young people in US, 31.1% of sexually experienced females and 45.0% of sexually experienced males reported six or more sex partners by age 21[18].

Although we found that older students were more likely than younger students to have had sex, those reporting multiple partners were more likely to have started having sex at younger age than those with only one partner. Other investigators have found that younger age is a risk factor for multiple partners [18]. Michelle Rotermann analyzed 2003 Canadian community health survey data and found that higher proportions of youth aged 15 to 17 and 18 to 19 had had intercourse with multiple partners in the past year, compared with 20- and 24-year-olds, thinking this difference may reflect a tendency toward longer-term, monogamous relationships at older ages [32]. Earlier initiation of sexual intercourse among youth is associated with greater frequency of sexual activity, larger number of sex partners, the advent of teen pregnancy at younger ages, and increase in frequency of STDs in youth [33]. In this study, we found that those who first had sex at age 18 or younger were more likely to have multiple sex partners than females who first had sex at age 19 or older, thereby significantly increasing their risk of HIV/STDs and pregnancy. This suggests that females should be targeted with preventive interventions as teens (before college) to discourage premature initiation of sexual activity.

It was interesting that a higher score of sex-related knowledge distinguished students who did and did not report having sex. Because this study was cross-sectional, we cannot discern if greater knowledge of sex increased the likelihood of having sex, or if having sex made one more knowledgeable about it. However, the overall low scores on the sex-related knowledge scale is cause for concern, with less than 10% of the sample scoring 60 or above on a 100-point scale (a percentage that would be ranked a D or F by most teachers). Thus, there is clearly a role for more sex education in China.

Other researchers have found that attitudes to sex have an enormous influence on sexual behavior [34]. Our findings confirm that students holding an open attitude to sex are more likely to engage in sexual activity [35,36]. Students who agree or accept multiple sex partner behavior are 3 times more likely to report more sex partners. Peer influences are important, and students whose friends live with boyfriends and who work at places of entertainment (where alcohol and sex are likely present) are 2 times more likely to report more sex partners. Although it is difficult to change attitudes and peers norms, interventions need to address both. Curricula that include opportunities for discussion, role play, and practice of refusal skills have shown promise [37,38].

Our finding also suggested that masturbation is a risk factor of multiple sex partner behavior among female undergraduates. In a study on masturbation and premarital sexual intercourse among college women [39], Davidson JK found masturbation was associated with multiple sex partners and unprotected sexual intercourse. In China, few studies have been conducted to investigate this behavior.

We also identified several variables that differentiate students who do and do not engage in premarital sex, and additional interventions can be developed targeting these students. For example, students coming from richer families and from families of divorce can be identified upon admission to school. Teachers can be encouraged to refer students with low academic performance or who seem depressed for special counseling. Campus policies can be established that restrict students from taking jobs at places of entertainment.

Although predictors of condom use is the subject of a future paper, it is critical to keep in mind that 30% of women having sex and 40% of women reporting multiple sex partners were inconsistent in their condom use (either reporting never or seldom using condoms when having sex). Increasing knowledge, attitudes, and skills associated with condom use are important objectives of a sex education intervention in China, and condoms should be easily accessible to students on college campuses.

As noted earlier, this study was limited by its cross-sectional design. Determining causality must be based on future longitudinal research. Secondly, sexual behavior is a sensitive subject and socially unacceptable in Chinese cultural settings [40], thus it is possible that students underreported their behaviors. However, by ensuring privacy during the completion of the questionnaire and using the anonymous self-administered survey, ever attempt was made to minimize this bias.

## Conclusion

This is the first study that examines multiple sex partner behavior and its risk factors among Chinese female undergraduates. It also updates estimates of sexual activity in this population. Our findings provide guidance to health educators for developing effective and feasible intervention strategies targeting female undergraduates who are at increased risk for HIV/STDs infection. First, female undergraduates should be targeted with preventive interventions when they are in middle schools so as to discourage their sexual initiation. Second, sex education must include material that will increase sex-related knowledge. Third, interventions must attempt to address attitudes and peer influences, and aim to increase consistent use of condoms among women who chose to engage in premarital sex. Forth, college campuses can institute programs for female students from richer families, from families of divorce, who are doing poorly in school, and/or appear depressed, as these factors are associated with risky behavior. A policy restricting female students from working at places of entertainment also should be considered.

## Competing interests

The authors declare that they have no competing interests.

## Authors' contributions

HY performed statistical analysis, interpreted the results and drafted the manuscript. WC and HW involved in interpretation of the results and participated in preparation of the manuscript. YB participated in the design of the study and made contributions to the revisions of the manuscript. MZ involved in statistical analysis and interpretation of the results. SL conceived of the study, participated in its design, supervised all aspects of its implementation and interpreted results. KLB made contributions to statistical analysis, interpretation of the results and the edit of the manuscript. All authors read and approved the final manuscript.

## Pre-publication history

The pre-publication history for this paper can be accessed here:


